# Clinical consequences, treatment status, and parental awareness of traumatic dental injuries among high school students: a cross-sectional study in Cairo

**DOI:** 10.1186/s12903-026-09675-8

**Published:** 2026-09-05

**Authors:** Maha A. Elhousiny, Nada A. Salem, Mervat I. Fawzy, Mai M. Mahmoud

**Affiliations:** 1 Endodontic Department, Faculty of Dental Medicine for Girls, Al-Azhar University, Cairo, Egypt; 2 Department of Pediatric Dentistry and Dental Public Health, Faculty of dentistry- October 6 University, Cairo, Egypt; 3 Dentist at the Ministry of Health, Cairo, Egypt

**Keywords:** Traumatic dental injuries, Clinical consequences, Adolescents, Parental awareness, School-based study, Cairo, Egypt.

## Abstract

**Background:**

This study assessed the current clinical consequences and treatment status of previous traumatic dental injuries (TDIs) affecting permanent anterior teeth among adolescents aged 15–17 years in Cairo, Egypt, and evaluated parental awareness and knowledge regarding the emergency management of TDI.

**Subjects and methods:**

A school-based analytical cross-sectional study assessing treatment history was conducted among 2,000 secondary school students aged 15–17 years using multistage stratified sampling. Stage 1: Students underwent standardized clinical examinations, during which a diagnostic chart was completed to determine the prevalence of traumatic dental injuries (TDIs), with a collection of self-reported history on trauma, sociodemographic characteristics, cause and location of injury, treatment type, time elapsed since injury, and the activity at the time of trauma. Traumatized teeth were classified as either treated or untreated. Current clinical consequences were interpreted conceptually as persistent manifestations of the original injury, possible post-traumatic clinical findings, an indeterminate structural outcome, or a patient-reported impact. Tooth-level comparisons used binary logistic generalized estimating equations (GEE) clustered by student. Stage 2: A structured questionnaire to assess parental awareness of TDI management, treatment-seeking behavior and emergency knowledge among parents of traumatized students was distributed to the parents or legal guardians of students who met the inclusion criteria, along with written informed consent. Statistical analysis included descriptive statistics, bivariate analyses, and clustering-adjusted generalized estimating equations for tooth-level clinical findings.

**Results:**

Previous TDIs were confirmed in 340 students. A total of 350 traumatized teeth were recorded; 29 teeth were treated, and 321 were left untreated. Parental awareness questionnaires were completed by 240 parents. Clinical consequences were more frequent in untreated than treated teeth. Uncomplicated crown fracture, tooth discoloration, and esthetic complaints were the most common clinical findings. Sports and activity-related trauma contributed to nearly 70% of all recorded injury activities. Mouthguard use was uncommon. Parental knowledge of emergency management was limited.

**Conclusion:**

Most recorded TDIs remained untreated, which contributed to a higher occurrence of complications in the affected teeth. These findings highlight the need to raise parental awareness of early intervention and first-aid measures and to improve students’ awareness of the importance of mouthguard use during sports.

## Background

Traumatic dental injuries (TDIs) are a significant public health concern and frequently represent urgent clinical conditions requiring timely and appropriate management. Failure to provide prompt and evidence-based care may increase morbidity, complicate treatment, and impose substantial economic burdens on individuals and healthcare systems. The global prevalence of TDI ranges widely from 4% to 58%, with approximately one-quarter of school-aged children experiencing dental trauma [1–3]. Maxillary central incisors are the most commonly affected teeth due to their anterior position and incomplete root development. Such injuries may result in functional impairments in mastication and speech, as well as esthetic concerns that negatively affect psychological well-being and social interactions [4].

Sports- and activity-related incidents are major contributors to TDIs among children and adolescents. Participation in contact sports, including martial arts, basketball, and soccer, as well as falls and collisions during routine activities, significantly increases the risk of injury [5]. Many of these injuries are preventable through the use of protective devices, particularly custom-made mouthguards. However, compliance with mouthguard use remains low, highlighting the need for enhanced preventive strategies, stricter safety regulations, and targeted educational interventions for students, parents, and school personnel [6, 7].

The etiology of TDI is multifactorial, involving biological, behavioral, and environmental determinants. Epidemiological evidence indicates that males are more frequently affected than females, with a reported global male-to-female ratio of approximately 1.4:1 [4, 8, 9]. Anatomical risk factors such as increased incisal overjet (> 3.5 mm), inadequate lip coverage, and anterior open bite are strongly associated with an increased risk of trauma to anterior teeth. Socioeconomic status also plays a role, with higher prevalence rates often observed among children attending public schools and those from lower-income backgrounds [10].

Timely intervention is a critical determinant of prognosis, particularly in severe cases such as avulsion and complicated crown fractures. As many TDI occur in home or school settings, parents, teachers, and coaches are often the first responders. Appropriate emergency management at the site of injury can significantly improve clinical outcomes. Nevertheless, delays in seeking treatment are common and are often attributed to a lack of awareness regarding the urgency of care or the misconception that minor dental injuries do not require professional attention. Improving knowledge of emergency management among caregivers and educators is therefore essential to enhance outcomes [6, 11].

Despite extensive research on the prevalence and risk factors of TDI, there is a lack of school-based studies evaluating the clinical consequences of untreated TDI among older adolescents. Furthermore, limited evidence exists regarding the relationship between these clinical consequences and factors such as parental awareness, knowledge of emergency management, and awareness of preventive measures, including mouthguard use, among adolescents aged 15–17 years. Addressing these gaps is essential for developing targeted public health interventions and improving treatment-seeking behavior in this population. In this study, the term “clinical consequences” is used as an umbrella descriptive term encompassing persistent manifestations of the original injury, possible post-traumatic clinical findings, trauma-related structural outcomes, and patient-reported impacts.

This study aimed to assess the presence of current clinical consequences of previous TDIs as the primary outcome. The treatment status of traumatized teeth, parental awareness, knowledge of emergency management, and time elapsed since trauma—affecting permanent anterior teeth among adolescents aged 15–17 years—were assessed as secondary outcomes.

## Materials and methods

### Study design

First stage: This is an analytical school-based cross-sectional study that included a retrospective inquiry component, designed to evaluate clinical consequences and treatment status of previous TDIs in permanent anterior teeth among adolescents. The retrospective component refers specifically to the collection of self-reported history regarding the age at injury, cause of trauma, time elapsed since injury, and type of treatment; these data were collected at a single point in time through a structured clinical examination. The study was conducted in high schools across Cairo, Egypt.

Second stage: Parental awareness of traumatic dental injury management—comprising treatment-seeking behavior and emergency knowledge—was surveyed using a self-administered structured questionnaire. The questionnaire was adapted and modified from the instrument reported by Salem et al. [12]. In the source study, content validity was assessed through review by two specialists in pediatric dentistry and public health, and the parent/teacher evaluation scale demonstrated acceptable internal consistency (Cronbach’s α = 0.814). Content validity of the adapted, TDI-specific version used in the present study was confirmed by an expert panel comprising a specialized Pedodontist and specialized Endodontists (members of the Al-Azhar Trauma Centre), who reviewed all items for relevance and clarity prior to collection. The adapted questionnaire was subsequently pilot tested with 20 parents, independent of the 240 parents included in the main analysis, to assess comprehensibility; no substantive revisions were required.

### Sample size calculation

The required sample size was estimated using the Epi-Info program (version 3.3.2), assuming a TDI prevalence of approximately 10–15% based on a previously published epidemiological study [4], a 95% confidence level, and a 5% margin of error. The minimum calculated sample size was 1,428 students. This was increased to 2,000 to account for non-response, potential exclusions, and to enhance statistical power, and a correction factor of 1.5 was applied to accommodate the multistage stratified sampling design.

### Study population and eligibility criteria

The target population comprised male and female students aged 15–17 years enrolled in secondary schools in Cairo Governorate and their parents/guardians. The following eligibility criteria were applied.

### Inclusion criteria

Students were eligible if they were aged 15–17 years and enrolled in the selected secondary schools; had fully erupted permanent anterior teeth (upper and lower incisors and canines); showed clinical evidence or reported a history of previous TDIs affecting one or more permanent anterior teeth, with the injury having occurred at least six months before the examination date; and had parents who agreed to participate. Written informed consent and a completed self-administered questionnaire were obtained from the parent or legal guardian, together with written assent from the student.

### Exclusion criteria

Students were excluded if they were undergoing orthodontic treatment with appliances that prevented accurate clinical assessment of the anterior teeth or occlusal variables; had missing anterior teeth due to caries, congenital absence, or any cause unrelated to dental trauma; presented developmental enamel defects that could be clinically misidentified as trauma-related fractures; had sustained acute trauma less than six months before the examination date; or declined participation in the clinical examination or questionnaire.

### Sampling procedure

The official sampling frame was obtained from the Cairo Educational Region through the relevant educational authorities. It comprised 112 secondary schools enrolling 24,912 students across five educational administrations: East Cairo (31 schools), West Cairo (17 schools), North Cairo (15 schools), South Cairo (27 schools), and Central Cairo (22 schools). Schools were selected proportionally from the five administrations and included governmental, government-aided, and private schools to provide broad geographic and socioeconomic coverage across Cairo Governorate. Administrative approval to access the schools was obtained from the Cairo Governorate Directorate of Education, which provided the official sampling frame and authorized access to the selected schools. Formal study-information letters were subsequently submitted to the administrations of the selected schools, and approval was obtained from the respective school principals before student recruitment and clinical examination. All eligible students in the selected schools were approached for participation. The design was intended to provide broad coverage; however, because school-level selection probabilities and sampling weights were not available for design-adjusted analysis, the findings should not be interpreted as a fully design-weighted representative estimate of all adolescents in Cairo Governorate.

### Data collection instruments

1. Student:

A- Questionnaire

A structured, closed-ended questionnaire was developed in Arabic, the native language of the study population. The questionnaire items and response categories were derived from variables and classification schemes commonly reported in previous school-based surveys of TDI and sports-related trauma [5, 13, 14]. Before the main study, the questionnaire was pilot tested with 20 students with characteristics comparable to those of the target population. The pilot assessment evaluated item clarity, comprehensibility, suitability of the response options, question sequence, feasibility of administration, and the time required to obtain the trauma history. The pilot study indicated no need to modify the proposed methods. The pilot participants were not included in the final study sample. The questionnaire comprised two main sections:

- Section 1 — Sociodemographic characteristics:
  - Age, sex, school name.
  - Residential area (urban/rural)
  - Father’s educational level (educated /non-educated / unknown- deceased)
  - Mother’s educational level (educated /non-educated / unknown- deceased)
  - Maxillary incisal overjet measured with a CPI probe in centric occlusion (categorized as ≤ 3 mm / >3 mm)

- Section 2 — Etiology, location, activity, treatment, and preventive awareness among students with traumatic dental injuries
  - Etiology of Trauma (Fall-Sports / play-related injury- Collision with object or person -Bicycle/road traffic accident-Fight / physical assault-Unknown / could not recall)
  - Place where the injury occurred (home/school/sports club or playground/street or outdoor/other)
  - Activity during which the injury occurred (Organized sports activity- School play/running- Cycling/scooter activity- Fight / physical assault- non-activity-related accident)
  - Time Elapsed Since Trauma (6–12 months- >1–2 years- >2–3 years->3 years)
  - Type of treatment received, if any (Composite restoration - fragment reattachment- Endodontic treatment- Extraction)
  - Self-Medication as Sole Response to Injury (Yes — used analgesics/antibiotics without dental visit- No self-medication)
  - Mouthguard Use During Sports (Yes- No)
  - Supervision at Time of Injury (Supervised by teacher/coach/parent -Not supervised -Unknown)

- B- Clinical Examination

- Section − 3 (Current clinical sequelae among previously traumatized permanent anterior teeth by treatment status)

Clinical examinations were conducted by a single calibrated examiner under standardized conditions. The examiner received theoretical training at the Faculty of Dentistry, Al-Azhar University, covering Andreasen’s epidemiological classification of TDI, operational definitions of the recorded clinical findings, standardized examination procedures, and completion of the clinical recording form. Before the start of data collection, clinical calibration was performed against an experienced pediatric dentistry specialist using 20 TDI-positive students who were not included in the main study; inter-examiner κ for this calibration exercise ranged from 0.71 to 0.94 across the categorical clinical variables assessed. Throughout the data collection period, intra-examiner and inter-examiner reliability were further assessed in a randomly selected subsample comprising 5% of the total study sample (*n* = 100), with repeat examinations conducted after a two-week interval. Cohen’s kappa coefficients for this ongoing reliability assessment ranged from 0.80 to 0.92 for intra-examiner agreement and 0.79–0.89 for inter-examiner agreement across all clinical variables, indicating substantial to almost perfect agreement. Reliability estimates for each clinical variable are reported separately in Supplementary Table S1 and interpreted using Landis and Koch’s conventional categories [15].

Students were examined in their classroom seats during school hours in the presence of natural daylight, supplemented by a portable light source to ensure adequate illumination. Standard infection control measures were observed, including the use of disposable diagnostic instruments for each student. A student was considered TDI-positive when a history of trauma was reported or when fracture, discoloration, trauma-related restoration, or trauma-related tooth loss was identified clinically. Injuries were classified according to Andreasen’s epidemiological criteria and the 2020 International Association of Dental Traumatology (IADT) guidelines. Treatment status was recorded for each traumatized tooth as treated or untreated. Students with signs requiring urgent attention, including acute swelling, abscess, or a sinus tract, were referred for prompt dental assessment through the Al-Azhar Trauma Centre for emergency dental care, independent of their inclusion status in the study, in accordance with IADT emergency management principles [16–18].

For this study, the term “clinical consequences” is used as a broad descriptive term for current trauma-related findings identified at examination. It includes persistent manifestations of the original injury, possible post-traumatic clinical findings, and other trauma-related outcomes, and does not imply that every recorded finding represents a confirmed secondary complication. For each traumatized tooth, current findings were separated conceptually into: (a) persistent injury/structural findings - uncomplicated crown fracture, complicated crown fracture, and trauma-related tooth loss; and (b) possible post-traumatic signs or sequelae - discoloration, sinus tract/swelling, abnormal mobility, pain/sensitivity, and tenderness to percussion.

- 2. Parents: (Evaluation of knowledge level)

Parental awareness of emergency TDI management was assessed using a structured questionnaire completed by parents or guardians of students with confirmed injuries [12–16, 19–21]. Of the 340 eligible parents, 240 returned usable questionnaires (response rate: 70.6%); the remaining 100 were excluded from awareness-related analyses.

The adapted, seven-item questionnaire comprised two sections.

Section A — Treatment-Seeking Behavior and Prior Education comprised two items assessing the parent’s immediate response when their child experienced a dental injury, and whether the parent had previously received any education about emergency management of dental trauma. These items were analyzed individually as categorical variables and were not included in the knowledge score.

Section B — Emergency Knowledge of Dental Trauma Management assessed emergency knowledge using five items, each scored 1 for a correct response or 0 for an incorrect or unknown response (range: 0–5). Correct responses were defined according to current IADT guidelines: prompt referral to a dentist, appropriate handling of an avulsed tooth, use of milk as a storage medium when immediate replantation is not possible, and retrieval and preservation of any fractured tooth fragment [16]. Section B was scored as a summative scale; because no validated cutoff for parental TDI awareness has been established for this population, scores of 0–3 were classified a priori as inadequate and scores of 4–5 as adequate.

### Questionnaire administration and informed consent

Before clinical examination and questionnaire administration, eligible students and their parents or legal guardians received information about the study objectives, procedures, the type of information to be collected, confidentiality, the voluntary nature of participation, and the right to withdraw without penalty. Written informed consent was obtained from a parent or legal guardian, and written assent was obtained from each participating student. Separate structured questionnaires were administered to students and responding parents or legal guardians. The investigator remained available during questionnaire completion and clinical screening to clarify questions without directing or influencing participants’ responses.

### Statistical analysis

Data were entered and analyzed using IBM SPSS Statistics, version 21.0 (IBM Corp., Armonk, NY, USA). Categorical variables are presented as frequencies and percentages; the continuous variable age is presented as mean ± standard deviation. Differences between students with and without TDI were examined using the independent samples t-test for age (after confirmation of approximate normality by inspection of histograms and the Shapiro–Wilk test) and the chi-square test for categorical variables. Fisher’s exact test was applied when any expected cell count was less than five. The tooth was the unit of analysis for current clinical findings (350 teeth in 340 students). Because 10 students contributed two teeth, each finding was modelled separately using binary logistic generalized estimating equations with Student ID as the clustering variable, a binomial distribution, logit link, independence working correlation, and robust sandwich covariance [14]. Treatment status was the sole covariate distinguishing treated from untreated teeth, with treated teeth as the reference. Odds ratios (OR), 95% confidence intervals (CI), and robust p values are reported. When a finding had zero events in treated teeth, the OR was reported as not estimated and Fisher’s exact p values were reported.

## Results

### Participant characteristics and prevalence of TDI

A total of 2000 students aged 15–17 years were examined. Previous TDIs to permanent anterior teeth were identified in 340 students, giving an overall prevalence of 17%. Students with TDI were younger than those without TDI (15.29 ± 0.58 vs. 15.84 ± 0.90 years; *p* < 0.001). TDIs were more frequent among 15-year-old students than among older students, with 156 cases (45.9%) occurring in this age group. Male students represented 294 of the TDI cases (86.5%), compared with 658 students (39.6%) in the non-TDI group (*p* < 0.001). Rural residence was also more frequent among students with TDI than those without TDI (306, 90.0% vs. 688, 41.4%; *p* < 0.001). Increased overjet > 3 mm was observed in 36 students with TDI (10.6%) and 89 students without TDI (5.4%) (*p* < 0.001) (Table 1).

**Table 1:** Association between traumatic dental injuries and sociodemographic characteristics among examined students (*n* = 2000)

Sociodemographic Variable	TDI Present *n* = 340 (%)	TDI Absent *n* = 1660 (%)	*p*-value
Age, mean ± SD (years)	15.29 ± 0.58	15.84 ± 0.90	< 0.001*
Age group			
15 years	156 (45.9%)	520 (31.3%)	
16 years	112 (32.9%)	610 (36.7%)	< 0.001*
17 years	72 (21.2%)	530 (31.9%)	
Sex			
Male	294 (86.5%)	658 (39.6%)	< 0.001*
Female	46 (13.5%)	1002 (60.4%)	
Residence			
Urban	34 (10.0%)	972 (58.6%)	< 0.001*
Rural	306 (90.0%)	688 (41.4%)	
Father’s Education			
Non-Educated	4 (1.2%)	49 (3.0%)	< 0.021*
Educated	316 (92.9%)	1541 (92.8%)	
Unknown / not stated	20 (5.9%)	70 (4.2%)	
Mother’s Education			
Non-Educated	72 (21.2%)	970 (58.4%)	< 0.001*
Educated	220 (64.7%)	650 (39.2%)	
Unknown / not stated	48 (14.1%)	40 (2.4%)	
Maxillary Overjet			
≤ 3 mm	304 (89.4%)	1571 (94.6%)	< 0.001*
> 3 mm	36 (10.6%)	89 (5.4%)	

Data presented as n (%). Age compared using independent samples t-test; categorical variables compared using Chi-square test p-values represent comparison between TDI-present and TDI-absent groups

^*^Statistically significant at *p* < 0.05

### Pattern and characteristics of traumatic dental injuries

The 340 students contributed 350 traumatized permanent anterior teeth; 10 students had two traumatized teeth. At the tooth level, 29 teeth had received definitive treatment, and 321 remained untreated. Among treated teeth, composite restoration or fragment reattachment was most frequent (17/29, 58.6%), followed by endodontic treatment (11/29, 37.9%) and extraction (1/29, 3.4%) (Table 2).

**Table 2:** Etiology, location, activity, treatment and preventive awareness among students with traumatic dental injuries (*n* = 340)

Variable	Category	*n*		%
Etiology of Trauma *(n = 340)*				
	Fall	88		25.9%
	Sports / play-related injury	128		37.6%
	Collision with object or person	49		14.4%
	Bicycle /road traffic accident	28		8.2%
	Fight / physical assault	16		4.7%
	Unknown / could not recall	31		9.1%
Place of Trauma *(n = 340)*				
	Home	124		36.5%
	School	122		35.9%
	Sports club / playground	44		12.9%
	Street / outdoor	50		14.7%
Activity at Time of Trauma *(n = 340)*				
	Organised sports activity	128		37.6%
	School play / running	112		32.9%
	Cycling / scooter activity	32		9.4%
	Fight / physical assault	16		4.7%
	Non-activity-related accident	52		15.3%
Time Elapsed Since Trauma *(n = 340)*				
	6–12 months	58		17.1%
	> 1–2 years	77		22.6%
	> 2–3 years	91		26.8%
	> 3 years	114		33.5%
Type of Treatment Received *(among treated teeth only*, *n = 29)*				
	Composite restoration / Fragment reattachment	17		58.6%
	Endodontic treatment	11		37.9%
	Extraction	1		3.4%
Mouthguard Use During Sports *(n = 340)*				
	Yes	2	0.6%	
	No	338	99.4%	
Supervision at Time of Injury *(n = 340)*				
	Supervised by teacher / coach / parent	66	19.4%	
	Not supervised	196	57.6%	
	Unknown	78	22.9%	

Data presented as n (%). Total n = 340 students for all injury and prevention variables unless stated. Type of treatment reported among treated teeth only (n = 29); treatment percentages use n = 29 as denominator. Supervision: “Unknown’’ (n = 78, 22.9%) reflects cases where supervision status could not be confirmed by questionnaire response

### Etiology, place, and time elapsed since injury

Sports/play-related injury was the most frequently reported etiology of trauma (128, 37.6%), followed by falls (88, 25.9%) and collision with an object or person (49, 14.4%). The most common places of trauma were home (124, 36.5%) and school (122, 35.9%). Regarding activity at the time of trauma, organized sports activity was reported by 128 students (37.6%), followed by school play or running in 112 students (32.9%). More than half of the students reported that the injury had occurred more than two years earlier, including 91 students (26.8%) with injuries > 2–3 years previously and 114 students (33.5%) with injuries > 3 years previously (Table 2).

### Treatment-seeking behavior and parental awareness

Only 22 students (6.5%) reported self-medication with analgesics, antibiotics, or pharmacy consultation as the sole response to injury without definitive dental treatment. Mouthguard use during sports was very low, with only 2 students (0.6%) reporting use. Most injuries occurred without confirmed supervision; 196 students (57.6%) were not supervised at the time of injury, while 66 students (19.4%) were supervised, and 78 students (22.9%) had unknown supervision status (Table 2).

Parental-awareness data were available for 240 responding parents. Only 14 parents (5.8%) reported taking the child to a dentist within 24 h of injury, while 186 parents (77.5%) reported that no professional care was sought. Previous education about emergency management of dental trauma was reported by only 5 parents (2.1%). Knowledge of avulsion was limited; 63 parents (26.3%) knew the term, 32 (13.3%) selected the correct immediate response for a knocked-out permanent tooth, and 25 (10.4%) identified milk as the recommended storage medium. However, 152 parents (63.3%) stated that they would search for a broken tooth fragment, and 83 (34.6%) knew that a dentist could reattach a fragment (Table 3).

**Table 3:** Parental awareness of traumatic dental injury management: treatment-seeking behavior and emergency knowledge among parents of traumatized students (n = 240; response rate = 70.6%)

Question	Response Option	n	%	Correct (IADT)
Question-A Treatment-Seeking behavior and Prior Education				
Q1. What did you do immediately when your child experienced a dental injury?	Took the child to a dentist within 24 hours	14	5.8%	
	Took the child to a dentist after delay (>24 hours)	30	12.5%	
	Visited a general physician or hospital emergency room	10	4.2%	
	Did not seek any professional care	186	77.5%	
Q2. Had you ever received any education about emergency management of dental trauma before this injury?	Yes	5	2.1%	
	No	235	97.9%	
Section-B Emergency Knowledge of Dental Trauma Management				
Q3. Do you know what the term ‘Avulsion’ means in the context of dental injuries?	Yes, I know what avulsion means	63	26.3%	✔
	No, I do not know	177	73.8%	
Q4. What would you do if your child’s permanent front tooth was completely knocked out?	Wash gently and replant immediately or store correctly	32	13.3%	✔
	Wrap in dry, clean gauze until reaching the dentist	174	72.5%	
	Discard the tooth – it cannot be saved	32	13.3%	
	Scrub the tooth clean before handling	2	0.8%	
Q5. What is the best medium to store a knocked-out tooth for transport?	Milk (IADT-recommended storage medium)	25	10.4%	✔
	Physiological saline / sterile water	69	28.8%	
	Disinfectant solution	54	22.5%	
	Tissue or dry gauze	39	16.3%	
	Baby’s mouth (buccal sulcus)	23	9.6%	
	Cotton pad or dry container	20	8.3%	
	Tap water	6	2.5%	
	Ice	4	1.7%	
Q6. Would you search for a broken tooth fragment at the site of the accident?	Yes, I would search for the fragment	152	63.3%	✔
	No, I would not look for it	88	36.7%	
Q7. Can a dentist reattach a broken tooth fragment to a traumatized tooth?	Yes, it can be reattached by a dentist	83	34.6%	✔
	No, it cannot be reattached	157	65.4%	

*n* = 240 parents of students with clinically confirmed traumatic dental injuries who agreed to complete the questionnaire (response rate: 240/340 = 70.6%)

Q1 was phrased retrospectively: ‘what did you do when your child’s injury occurred?’ to capture actual behavior, not hypothetical intent

✔ = Correct response according to International Association of Dental Traumatology (IADT) guidelines

### Current clinical consequences of previous TDIs

Clinical sequelae were assessed at the tooth level among 350 traumatized teeth. An uncomplicated crown fracture was the most common finding overall, affecting 176 teeth (50.3%). Esthetic complaint was recorded for 202 teeth (57.7%), tooth discoloration for 67 teeth (19.1%), pain/sensitivity for 74 teeth (21.1%), and complicated crown fracture for 41 teeth (11.7%). Sinus tract/swelling, abnormal mobility, pain on percussion, and trauma-related tooth loss were less frequent. In clustered GEE analyses, untreated teeth had significantly higher odds of uncomplicated crown fracture (OR = 5.47, 95% CI 2.18–13.75, *p* < 0.001) and pain/sensitivity (OR = 8.24, 95% CI 1.18–57.64, *p* = 0.034) compared with treated teeth. Odds were elevated but not statistically significant for tooth discoloration, complicated crown fracture, and pain on percussion. Sinus tract/swelling and abnormal mobility occurred in zero treated teeth, excluding GEE estimation (Fisher’s exact *p* = 0.610 and *p* = 0.392, respectively). (Table 4).

**Table 4:** Current clinical sequelae among previously traumatized permanent anterior teeth by treatment status, with clustering-adjusted GEE odds ratios (tooth-level analysis; treated *n* = 29, untreated *n* = 321, total *n* = 350)

Clinical Sequela	Treated teeth *n* = 29, n (%)	Untreated Teeth *n* = 321, n (%)	Total Teeth *n* = 350, n (%)	† OR (95% CI)	Robust *p*-value
Tooth discoloration	3 (10.3%)	64 (19.9%)	67 (19.1%)	2.16 (0.66–7.09)	0.205
Uncomplicated crown fracture	5 (17.2%)	171 (53.3%)	176 (50.3%)	5.47 (2.18–13.75)	< 0.001
Complicated crown fracture	1 (3.4%)	40 (12.5%)	41 (11.7%)	3.99 (0.52–30.31)	0.182
Sinus tract/swelling	0 (0.0%)	11 (3.4%)	11 (3.1%)	*‡* NE	-
Abnormal mobility	0 (0.0%)	20 (6.2%)	20 (5.7%)	*‡* NE	-
Pain/sensitivity	1 (3.4%)	73 (22.7%)	74 (21.1%)	8.24 (1.18–57.64)	0.034
Pain on percussion	2 (6.9%)	35 (10.9%)	37 (10.6%)	1.65 (0.39–6.92)	0.492
Tooth loss due to trauma	1 (3.4%)	2 (0.6%)	3 (0.9%)	0.18 (0.02–2.01)	0.161

† ORs compare untreated with treated teeth and were estimated with separate binary logistic GEE models clustered by Student_ID, using robust covariance; treated teeth as reference. Accounts for the 10 students who each contributed two traumatized teeth

‡ NE = not estimable: zero events among treated teeth produced complete separation; GEE could not be fit. Fisher’s exact p-value reported instead

Significant p-value (< 0.05)

## Discussion

Dental injuries among secondary school students aged 15–17 years represent a significant health problem, as they can affect adolescents both physically and psychologically. This concern is particularly important given the increasing rate of complications associated with delayed treatment and the limited awareness among parents regarding proper treatment approaches. The principal finding of this study was a 17% prevalence of TDI (340 of 2,000 students) among adolescents aged 15–17 years in Cairo Governorate. These findings are consistent with a previous studies in Egypt that reported a prevalence range from 13.6% to 14.6% [20, 22]. A recent systematic review and meta-analysis among Arab children reported a 26% burden of TDI with wide country variation levels [8]. The prevalence also aligned with the global range of 6% to 59% reported by Lam’s 2016 systematic review [23]. These variations in prevalence may be attributed to differences in age group, study design and location.

Male students accounted for 86.5% of the TDI-positive group compared with 39.6% of students without TDI, closely paralleling the pooled estimates from the Arab-country meta-analysis (OR approximately 2.06). This is likely attributable to greater engagement in contact sports, higher physical activity, and lower risk aversion compared with females, as well as differing behavioral and risk-taking profiles [13, 24]. In addition, 15-year-old adolescents were well represented among TDI cases (45.9%), likely reflecting recent exposure to sports activities upon high-school entry. Clinically, a maxillary overjet greater than 3 mm was significantly more prevalent in the TDI group (10.6% vs. 5.4%; *p* < 0.001). This is consistent with a meta-analysis reporting an OR of approximately 1.78 for an overjet greater than 3.5 mm [8]. Another study reported an OR of 2.32 (*p* = 0.034) for increased overjet [24]. Rural residence was associated with TDI in 90.0% of cases. This finding is consistent with the studies showing that adolescents in rural areas have a greater exposure to unsupervised high-energy physical activity [22]. This hypothesis is supported by this study’s finding that 37.6% of dental injuries occurred during these sports or play-related activities.

Sports- and activity-related dental injuries in the current study involved organized sport (37.6%) and school play or running (32.9%), which together accounted for more than 70% of injury-related activities. These findings align with a recent epidemiological study in which physical activity and sports were cited as major etiological factors for TDI in adolescents [6]. Despite this, mouthguard use at the time of injury was reported by only two students (0.6%). However, the exact sports involved and whether the participants were engaged in contact or collision sports were not identified. Therefore, the observed 0.6% should not be interpreted as a mouthguard-compliance rate among eligible high-risk athletes. A school-based study found that only 3% of contact-sport participants reported consistent mouthguard use [14], while research in Sri Lanka reported that although 20.8% of players used mouthguards, 68.1% of those who sustained an injury were not wearing one at the time of the incident [5]. The protective benefit of mouthguards is well established: not wearing a mouthguard is associated with a 1.6- to 1.9-fold increased risk of orofacial injury [25], and multiple reviews have confirmed a protective effect for both hard and soft tissues [25–27]. Promoting their uptake in adolescent sports—particularly football, cycling, and running—requires a concerted effort involving coaches, physical education teachers, and parents. Only 19.4% of injuries occurred under adult supervision, underscoring the critical need for structured safety protocols in school and sports settings. A scoping review of school-based dental trauma interventions concluded that a lack of knowledge and limited training in dental first aid were universal among educators, and that educational programs yield the greatest gains in knowledge [26]. Integration of a dental trauma first aid program in the physical education curriculum represents a low-cost, highly effective strategy in decreasing traumatic dental injuries in the school environment.

At the tooth level, 29 of 350 traumatized teeth (8.3%) had received definitive treatment, while 321 teeth (91.7%) remained untreated. Comparable patterns appear in the literature: one study reported that 95.16% of 186 traumatized students had received no professional dental treatment [28], while a study in Goa found that 12.4% of affected children were unaware that professional care was an option [29]. Similarly, research in Amman, Jordan, found that only 13.6% of children with TDI were treated [30]. Collectively, these data indicate that most adolescent dental trauma remains clinically unmanaged, substantially increasing the risk of long-term sequelae. The parental questionnaire data offer insight into this treatment gap.

Among the 240 parents who completed the questionnaire, 77.5% reported that they did not seek any professional care after their child’s trauma. Only 5.8% of parents brought their child to a dentist within 24 h. This represents a lower proportion of timely care than the 17.1% reported in Jordan and contrasts with Shenzhen, China, where 98.1% of patients sought care within the first 24 h [31]. The lack of intervention is a critical barrier to a favorable TDI outcome, as the prognosis of traumatized teeth is strongly influenced by prompt management [4]. Furthermore, 97.9% of responding parents had never received any prior education about the emergency management of dental trauma. The reasons for this lack of treatment-seeking are likely multifactorial and may include persistent gaps in awareness. However, this finding cannot be attributed to parental educational level because the study did not assess the association between parental education and treatment-seeking behavior. Epidemiological literature suggests that TDI may not be perceived by the public as a condition requiring professional care, leading to underestimation of its importance unless acute symptoms are present [32]. In addition, minor fractures, such as enamel fractures, may not cause functional discomfort and may therefore contribute to delayed or absent care-seeking [29]. These findings highlight the urgent need for community-level educational programs and a school-based protocol aimed at enhancing parents’ knowledge regarding the importance of immediate dental evaluation following any traumatic injuries.

The tooth-level findings demonstrate a substantial burden of current trauma-related conditions among adolescents with previous TDI. An important conceptual distinction is required when interpreting these observations. Unrepaired uncomplicated and complicated crown fractures represent persistent structural manifestations of the original injury rather than secondary biological complications. By contrast, discoloration, sinus tract or swelling, abnormal mobility, pain or sensitivity, and tenderness to percussion may represent possible post-traumatic pulpal, periapical, or periodontal findings. However, these findings could not be considered diagnostically confirmed complications because radiographic examination, comprehensive pulp sensibility or vitality testing, and longitudinal follow-up were not performed.

The clustering-adjusted tooth-level analyses showed that untreated teeth were more likely to present with unrepaired uncomplicated crown fracture and pain/sensitivity. The latter finding may reflect dentine exposure, bacterial penetration through exposed dentinal tubules, or possible early pulpal involvement [33, 34]. Treatment decisions may have been influenced by initial injury severity, symptoms, access to care, and family behavior, all of which can confound comparisons between treated and untreated teeth. The study design cannot establish the temporal pathway from injury through treatment status to the current finding.

To reduce the prevalence of TDIs among adolescents, the study’s conclusions have a number of practical public health implications. First, targeted rather than universal prevention strategies are most likely to be cost-effective, given the high prevalence, marked male predominance (86.5%), rural concentration (90.0%), and sports-related etiology [1]. Adolescents aged 15–17 years should be enrolled in regular school-based dental screening programs, with a focus on rural schools, to detect the high number of untreated injuries and ensure prompt referral to dental services. Second, the much higher prevalence of increased overjet in the TDI group (10.6% vs. 5.4%) supports combining orthodontic evaluation with dental screening; because early interceptive treatment may reduce this risk, students with an overjet greater than 3 mm should be prioritized for referral. This study has several limitations. First, its cross-sectional design, together with the retrospective collection of injury history, precludes causal and temporal inference. Second, because more than half of the injuries were reported to have occurred more than two years before examination, information on the cause and place of injury, activity at the time of trauma, supervision status, elapsed time, and previous treatment was vulnerable to recall bias and misclassification. Third, the study was restricted to school-attending adolescents aged 15–17 years in Cairo Governorate, which may limit the generalizability of the findings to younger children, out-of-school adolescents, or populations in other geographic settings. Fourth, the clinical examination did not include radiographic assessment or formal pulp sensibility or vitality testing; therefore, pulp necrosis, apical periodontitis, root fracture, inflammatory or replacement resorption, and other internal post-traumatic sequelae could not be identified. Fifth, parental-awareness findings should be interpreted cautiously because only 240 of the 340 eligible parents returned usable questionnaires, raising the possibility of non-response bias. Sixth, the small number of treated teeth and the low frequency of several clinical findings in the treated group resulted in sparse data, wide confidence intervals, and limited capacity for adjusted analyses. Finally, because the number of treated cases and outcome events was limited, any regression-based associations should be considered exploratory. No multivariable regression was retained.

## Conclusion

Most of the recorded TDIs remained untreated, which was associated with a higher occurrence of complications in the affected teeth. These findings underscore the need to raise parental awareness of early intervention and first-aid measures and to improve students’ awareness of the importance of mouthguard use during sports.

## Recommendations

1. Implement comprehensive school-based dental trauma prevention and screening programs, particularly in rural areas, to enable early detection of TDIs, identify high-risk students (e.g., those with increased overjet > 3 mm), and facilitate timely referral to appropriate dental services through standardized referral pathways.
2. Strengthen education and awareness initiatives for parents, caregivers, and school personnel by providing evidence-based training on the prevention and emergency management of TDIs, including the immediate management of avulsed teeth, appropriate storage media, and the importance of seeking dental care promptly, in accordance with IADT guidelines.
3. Promote preventive policies within schools and sports settings by mandating or strongly encouraging the use of mouthguards during contact and high-risk sports, supported by collaboration between schools, sports organizations, and public health authorities to improve compliance.
4. Leverage digital health technologies to enhance public awareness, deliver educational interventions through digital platforms and social media, improve parental engagement, and support surveillance and large-scale data collection for monitoring traumatic dental injuries.
5. Prioritise future research by conducting interventional studies to evaluate the effectiveness of preventive and educational strategies, investigating barriers to timely treatment-seeking through qualitative research, and including younger children and out-of-school adolescents to improve the generalizability of findings.

## Data Availability

The datasets used or analysed during the current study are available from the corresponding author on reasonable request.

## Abbreviations

TDI: Traumatic dental injury
OR: Odds ratio
CI: Confidence interval
GEE: Generalized estimating equations
SD: Standard deviation
SPSS: Statistical Package for the Social Sciences
IADT: International Association of Dental Traumatology
